# Sexual and Reproductive Health Service Seeking Scale (SRHSSS): development, validity, and reliability

**DOI:** 10.1186/s12889-024-17867-6

**Published:** 2024-02-03

**Authors:** Dilem Özdal, Meral Demiralp

**Affiliations:** 1 Department of Health Management, Faculty of Health Sciences, European University of Lefke, Northern Cyprus, Mersin, TR-10 Turkey; 2 Department of Psychiatric Nursing, School of Nursing, European University of Lefke, Northern Cyprus, Mersin, TR-10 Turkey

**Keywords:** Sexual and Reproductive Health (SRH), Young adults, Service seeking scale, Health services

## Abstract

**Background:**

Young adults are a diverse group with diverse cares, choices and preferences in accessing and using Sexual and Reproductive Health (SRH) services, they should be at the center of the development of new managed models and solutions for the delivery of SRH services. The purpose of the study is to develop a valid and reliable measurement tool that can be used to determine the knowledge and attitudes of young adults about SRH and the barriers for accessing services, and to evaluate the attitudes and needs of young adults to receive SRH services.

**Methods:**

In this study, the questions of the scale were developed through literature review, focus group interview with 8 people and expert evaluation were made, and a pre-test application was also carried out. Exploratory factor analysis and reliability testing were performed with a sample of 458 young adults. The re-test reliability was performed with 220 participants who were reached one month after the first measurement. Principal component analysis was used to establish the construct validity. The reliability of the scale was assessed using the Cronbach's alpha value.

**Results:**

A 23-item scale has been developed to identify and evaluate young adults' thoughts, attitudes, and perceived barriers for accessing services regarding SRH. In the exploratory factor analysis conducted to examine the construct validity of SRHSSS, a four-factor structure was obtained that explained 89.45% of the total variance. The factor loadings of the scale items were found to vary between 0.78–0.97. At the same time, the Cronbach's alpha value of the scale is 0.90, indicates a good internal consistency.

**Conclusions:**

SRHSSS is a scale with sufficient validity and reliability to determine young adults' SRH Service Seeking.

## Background

United Nations has reported that 1.2 billion young people, or 16% of the world's population, are between the ages of 15 and 24, and mentioned that the number of youth will increase by nearly 1.3 billion [1]. World Health Organization (WHO), defines "Adolescents" as 10–19 age range and "Youth" as 15–24 age range [2]. Many people have their first sexual and reproductive experiences in adolescence and young adulthood. Adolescents and young adults (AYAs), defined here as those aged 15–24 years, have these experiences during a critical time for psychological, social, cognitive, and physical development. During this stage of growth, AYAs create their own identity and establish autonomy, responsibility, and independence. At the same time, they are negotiating interpersonal relationships with peers, friends, parents, and guardians [3].

Individuals in young adulthood are sexually active, and open to different lives and behaviors in terms of thought and action. Young adults are more at risk than other age groups, especially due to the adverse effects of sexually transmitted diseases (STDs) such as HIV/AIDS, frequently changing sexual partners, engaging in early sexual acts, and early pregnancy [4, 5]. Many social problems such as age inequalities among heterosexual partners, gender differences in sexual behavior norms, early marriage for girls and the likelihood of sexual oppression also threaten the SRH of this age group [6]. Therefore, young adults are the best age group that could receive education and services to prevent adverse cases such as unwanted pregnancies and STDs [7].

Difficulties in accessing contraception and safe abortion, and the risk of contracting STDs, including HIV, are major challenges in young adults' lives. Studies by Lancet Commissions report that 25 million unsafe abortions are performed worldwide each year, more than 350 million men and women need treatment for one of four curable STDs, and approximately 2 million people are newly infected with HIV. In addition, one in three women has experienced intimate partner violence or non-partner sexual violence at some point in their lives. In the same study, it was reported that almost all of the 4.3 billion people of reproductive age worldwide would have inadequate SRH services during their lifetime [8].

On the other hand, there are still difficulties for young adults in accessing SRH services and exercising their SRH rights due to social and cultural reasons. Young adults try to solve their SRH problems by referring to unreliable sources on the internet instead of using counselling and treatment services provided by official institutions [9]. The reason for this, is stated as unpleasant or judgmental attitudes of healthcare professionals in a cross-sectional study conducted by Decker et al. in 2021 [10]. Health care providers do not recognize the needs of young people and welcome them, especially the unmarried. Thus, healthcare professionals may present an unsupportive and judgmental attitude towards young adults, thereby creating an obstacle to service procurement. The data on unintended pregnancy and abortion, violence against women, and STDs/HIV/AIDS are all restricted by the persistent taboos surrounding the subject of sexuality. In addition, in SRH services; Various political, economic and sociocultural factors can restrict the provision of information and services [11–13].

In some societies around the world, sexuality is still described with expressions such as "secret, shameful, forbidden" and is seen as a taboo. Especially young people cannot access sufficient and accurate information about sexuality, sexual and reproductive health [14]. The majority of adolescents in school have inadequate SRH, necessitating their need for access to comprehensive sexual education, information and services. Providing these services is necessary to ensure that adolescents can access, understand, evaluate and apply good SRH information in their decision-making processes for their own health [15]. In their study, Jinping Lyu and his colleagues drew attention to the importance of the issue by reporting that especially female students in China behave conservatively in sexual attitudes and behaviors and that low levels of sexual knowledge contribute to risky behaviors among Chinese adolescents [14].

In recent years, new measurement tools have been developed to evaluate the empowerment of adolescents and young adults regarding sexual and reproductive health [3]. Additionally, measurement tools are being developed to assess young people's needs and problems [16].

Providing health and counseling services related to SRH in official institutions, will provide a reliable official resource that young people can refer to if they are faced with serious dangers such as gender inequality, early and unwanted pregnancies, HIV and other STDs [17–19].

When the studies that have been done so far on receiving health services related to SRH with young adults are examined; Although the results of studies on the importance of young adults receiving services on SRH have been reported; There is no measurement tool that can evaluate the demand and need for service procurement from an official institution. Given the above-mentioned reasons, it is young adults who have the most difficulties in SRH and seek health services [20, 21]; It is important to determine the SRH services they need, and therefore the need for a valid and reliable measurement tool is inevitable.

Measuring the knowledge of a group also allows comparison with other groups and their progress over time. Changes in groups often represent results in knowledge, attitudes, or behavior. Thus, one of the greatest challenges when measuring the knowledge, attitudes, or behavior of a group may be measuring when these outcomes are most likely to be evident and are most meaningful. Problems such as unwanted pregnancies, increase in sexually transmitted diseases, sexual exploitation, violence, and adolescent marriages, especially in adolescence and youth periods. It is a risky period in some societies because of this and it is very important to receive reliable and healthy service from official institutions regarding sexual and reproductive health [22, 23].

The purpose of the study is to develop a valid and reliable measurement tool that can be used to determine the knowledge and attitudes of young adults about SRH and the barriers for accessing services, and to evaluate the attitudes and needs of young adults to receive SRH services.

## Methods

**Fig. 1:**
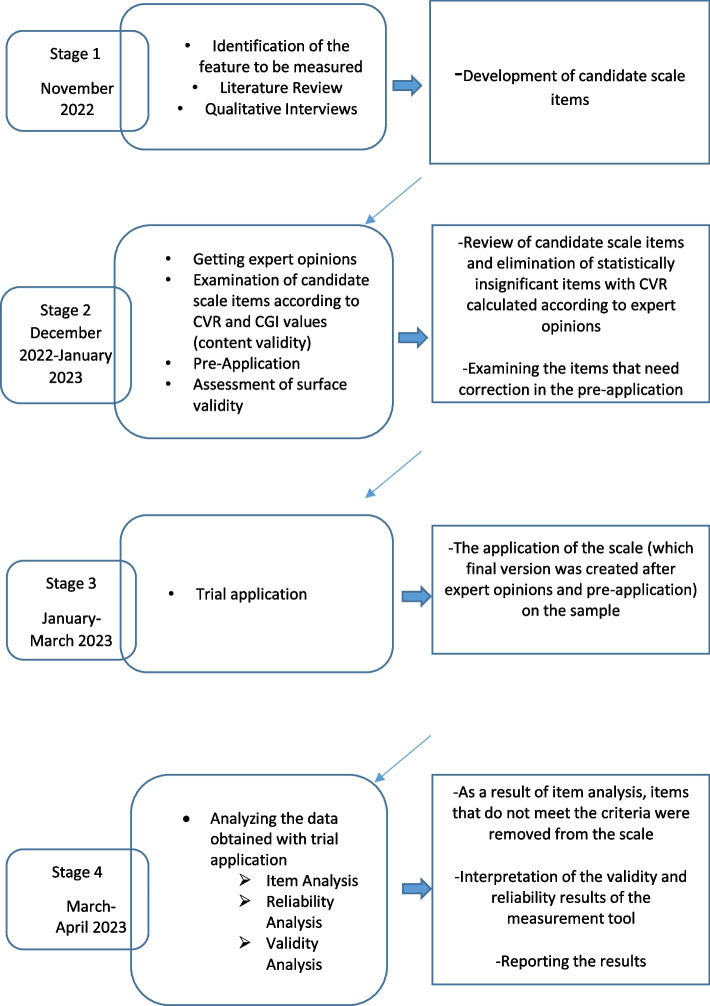
Steps followed for the design and the analysis of validity and reliability

This study was designed with a methodological study to develop a valid and reliable measurement tool in order to determine the knowledge and attitudes of young adults and the barriers for accessing SRH services, and also to evaluate the attitudes and needs of young adults to receive these services (Fig. 1).

### Steps followed in the scale development process

The feature to be measured in this study determined as “SRH service seeking”. When the literature on the subject was examined, no measurement tool could be found that could measure the SRH service seeking of young adults.

In this study, in determining the scope of the feature that will be measured, the literature on the subject was examined, and questionnaire questions were applied to 98 young adult individuals about the definition of sexual and reproductive health, whether they received information from any source about the subject, the ways of accessing information and whether the source of information was reliable. In addition to the participants participating in the survey, focus group interviews were conducted with 8 participants. In the focus group interview, 4 semi-structured open-ended questions were asked to the participants to convey their views and attitudes about SRH service seeking. These questions are, "What do you know about SRH?", "How do you access information about SRH?", "What do you think about whether the information you receive about SRH is reliable or not?" and “What do you think about getting services from an official institution on SRH?”. The focus group interview was conducted in a well-lit meeting room at the school where the researcher continued the work, with audio recording. The interview lasted 75 min. The interview was transcribed verbatim.

Qualifications for SRH service seeking were studied and statements that could be included in the scale were started to be written. While creating the scale items, attention was paid to include the description of the feature to be measured, to contain both positive and negative statements, and not to include more than one judgment/thought. In conclusion; For the draft version of the scale developed at this stage, a total of 23 items thought to be related to service seeking were determined. Expert opinion (Assoc. Prof. Dr. in Psychiatric Nursing, Assoc. Prof. Dr. in Obstetrics and Gynecology Nursing, Prof. Dr. in Obstetrics and Gynecology Nursing) was taken for the draft version of the scale and the results were evaluated. For the final version of the scale, the opinions of a language expert were taken before starting the application. The data obtained in this context were used to evaluate the content validity of the scale.

In the pre-application phase, the final version of the scale was applied to 15 young adults as a result of expert opinions. Thus, the intelligibility and applicability of the scale were investigated and it was determined that there were no incomprehensible expressions.

### Data collection form

The data collection form includes two parts. The first part consists of demographic information questions and status of SRH information. The second part consists of the “SRHSSS” (23 items), which was developed for this study and whose validity and reliability were examined.

SRHSSS is prepared in 3-point likert type. “I agree correctly” is scored as 2 points, “I disagree incorrectly” is scored as 0 points and “I don't know” is scored as 1 point. The lowest score that can be obtained in total is 0, and the highest score is 46. Low scores from the scale indicate that young adults do not feel ready and need to receive services related to SRH, while high scores indicate that young adults are ready and should receive services related to SRH. In order to assess the scale's invariance with respect to time, the re-test reliability was performed with 220 participants who were reached one month after the first measurement. For this study, the cut-off point of the scale was not calculated.

### Implementation

The target audience for the study was young adults in a province in Northern Cyprus, between the ages of 18 and 25, and the sample was made up of 458 of them who agreed to take part in the study. Due to the age of the participants, high schools and universities were visited and young adults who volunteered to participate were included in the study. Rourke and Hatcher reported that, it needs to obtain reliable results, the minimal number of participants providing usable data for the analysis should be the larger of 100 participants or 5 times the number of variables being analyzed. The calculation of the research sample of this study, from the sample size calculations foreseen for the scale development studies; (Number of Items):(Number of observations/person) ratio was used according to Rourke and Hatcher. Based on this calculation, a rate of 5–30 observations per item is suggested. SRHSSS items consist of 23-items. Although the five times the number of items on the scale equals 115, in this study the final sample has included 458 responses (1/20) [24].

### Data analysis

Age, gender, education level, and socioeconomic position were all examined using descriptive statistics. Prior to factor analysis, the KMO test and Bartlett's Sphericity test were used to determine if the sample size was adequate for factor analysis. Exploratory factor analysis and 'varimax' axis rotation principal component analysis, which are essentially used to group the related items in a cluster, were used to assess the construct validity of the scale.

In order to find out whether the established scale is reliable, internal consistency analysis and re-test reliability analysis were performed. Internal consistency analysis was used to get the Cronbach's Alpha values for each sub-dimension and all of the scale's statements. The correlation between the scores of the scale sub-dimensions obtained from the first and second applications was examined, and the Cronbach Alpha reliability coefficients were calculated for the re-test. The data was analyzed using IBM SPSS Statistics 20 software.

### Ethical dimension

The study was initiated to measure SRH services seeking of young adults between the ages of 18–25 in a province in Northern Cyprus. The purpose of the study was explained to the participants, and their participation was ensured to answer the scale questions on a voluntary basis.

All participants and their legal guardians provided informed consent and the European University of Lefke Scientific Research and Publication Ethics Committee approved the study protocol (25.10.2022/BAYEK015.010).

## Results

In this chapter; The descriptive features of the participants and the findings regarding the validity and reliability of the “SRHSSS” were presented. 

### Descriptive statistics

Table 1 presents the socio-demographic characteristics of the participants. The highest rate (22.7%) of the participants was 22 years old, 56.8% were women, 69.9% had a university-level education, 87.6% were single, and 64% had a low-income level. It is seen that 70.3% of them did not receive any information about SRH before, and 23.8% of those who received information used the Internet as an information source.

**Table 1:** Descriptive statistics of young adults (*n* = 458)

	n	%
**Age**		
18	18	3,9
19	29	6,3
20	27	5,9
21	27	5,9
22	104	22,7
23	86	18,8
24	84	18,3
25	83	18,1
**Gender**		
Female	260	56,8
Male	198	43,2
**Education Level**		
Middle School	3	0,7
High School	99	21,6
University	320	69,9
Graduate Education	36	7,9
**Marital Status**		
Married	57	12,4
Single	401	87,6
**Income Rate**		
Income Less Than Expense	293	64,0
Income Equals Expense	136	29,7
Income More Than Expense	29	6,3
**Status of Obtaining Information on SRH**		
Yes	135	29,7
No	323	70,3
**Information Source**		
Not Received Information	323	70,5
Family	14	3,1
Friends	12	2,6
Internet	109	23,8

Table 2 shows the results of factor analysis. The factor loadings of the scale ranged from 0.78 to 0.97. The difference between the two high load values of the items in the factor we obtained is more than 0.10. The eigenvalue of Factor 1 is 11.0, which accounts for 47.9% of the variation. The eigenvalues of Factor 2 are 5.26, which accounts for 22.8% of the variance, Factor 3 is 2.57, which accounts for 11.18% of the variance, and Factor 4 is 1.71, which accounts for 7.44% of the variance. The explained total variance is 89.45%. In this study, the item eigenvalues of the SRHSSS, which was created to determine the service seeking of the participants about SRH, were found to be between 0.78 and 0.97, that is, above 0.66, and the item load values were determined to be high and this result means that the SRHSSS items together measure a concept-structure-factor. Thus, SRHSSS is considered to be a scale with sufficient validity values to be used with young adults.

**Table 2:** Factor loadings and values of variance explained by SRHSSS

Factor 1	Factor 2		Factor 3		Factor 4
**Individual Considerations in Service Seeking**	**Individual Attitudes in Service Seeking**		**Education Need in Service Seeking**		**The Need for Institutional Support in Service Seeking**
ICSS 1 (0,91)	IASS 12 (0,78)		ENSS 18 (0,88)		ISSS 22 (0,95)
ICSS 2 (0,93)	IASS 13 (0,96)		ENSS 19 (0,92)		ISSS 23 (0,95)
ICSS 3 (0,93)	IASS 14 (0,97)		ENSS 20 (0,93)		
ICSS 4 (0,91)	IASS 15 (0,97)		ENSS 21 (0,91)		
ICSS 5 (0,82)	IASS 16 (0,97)				
ICSS 6 (0,90)	IASS 17 (0,96)				
ICSS 7 (0,92)					
ICSS 8 (0,92)					
ICSS 9 (0,94)					
ICSS 10 (0,90)					
ICSS 11 (0,91)					
**Eigen Value**	11,0	5,26	2,57	1,71	
**Variance Explained**	47,94	22,88	11,18	7,44	
**The Total Variance Explained**	89,45				

### Validity of SRHSSS

In this study, the KMO coefficient was found to be 0.88 and the Barlett sphericity test χ2 value = 19379.193 and *p* < 0.000. These results show that the data are suitable for factor analysis and the normality of the scores. In order to examine the factor structure of SRHSSS, exploratory factor analysis and Varimax with Kaiser Normalization test rotated principal component analysis were applied.

“The first subdimension, which is “Individual Considerations in Service Seeking”, of the scale includes the importance of SRH in terms of general health, well-being and quality of life of individuals and their thoughts on the necessity of obtaining basic information. It also reflects their thoughts on the importance of getting services from an official institution related to SRH, having regular health check-ups, protecting the person from risky situations and STDs, and using effective/healthy prevention methods.

The second subdimension, which is “Individual Attitudes in Service Seeking”, of the scale reflects the individual readiness and attitudes of individuals regarding SRH to talk with the health personnel, where they can get counseling in an official institution, to be informed and to express their problems.

The third subdimension, which is “Education Need in Service Seeking”, of the scale reflects the thoughts that the age-appropriate education about SRH, which can be given by the family and an official institution, protects individuals from encountering undesirable health problems, prevents them from accessing unreliable sources about this issue, and enables them to make conscious choices.

The fourth subdimension, which is “The Need for Institutional Support in Service Seeking”, of the scale includes thoughts on a counseling support line that can be established by an official institution that aims to provide individuals with access to accurate and reliable information, rather than using unreliable sources about SRH.”

### Reliability of SRHSSS

For this research; the internal consistency of the questionnaire (SRHSSS), which was created to determine the service seeking of the participants about SRH, was examined and the reliability coefficient Cronbach's Alpha value was determined as 0.90. The obtained value showed us that, the SRHSSS, which was developed and used as a determinant tool for SRH service seeking for this study, reflects real differences at the rate of 90% and is statistically reliable (*p* < 0.000).

Examining Table 3, The test and the re-test were shown to have a strong, favorable, and significant relationship, *r* = 0.713, *p* < 0.001. Accordingly, it can be said that as the scores in the first application increase, the re-test scores also increase. Table 3 also shows the item-total correlations of the factors.

**Table 3:** Reliability coefficients of SRHSSS and the factors

Scale and Sub-Dimensions	Number of Items	Item-Total Correlations	Test 1 Cronbach Alfa	Re-test Cronbach Alfa	Re-test Correlation
SRHSSS	23	0,410–0,771	0,902	0,901	0,713
Individual Considerations in Service Seeking	11	0,516–0,623	0,985	0,988	0,515
Individual Attitudes in Service Seeking	6	0,683–0,771	0,975	0,992	0,783
Education Need in Service Seeking	4	0,461–0,519	0,967	0,976	0,508
The Need for Institutional Support in Service Seeking	2	0,410–0,419	0,959	0,989	0,610

These results can be interpreted as the validity of the items in the scale is high and it distinguishes the participants in terms of seeking services on SRH; indicates that the discriminative power of the items is sufficient. When the first test and re-test cronbach alpha values are examined, the general values for both applications show 0.90 and these tests reflect that they are 90% reliable.

Table 4 presents the correlation values between each item score in the SRHSSS and the total scale score, as well as 27% lower upper t values. When the table is examined, the item-total correlations for every item on the scale range from 0.410 to 0.771, as can be observed and the 't' values are significant (*p* < 0.001). The service-seeking scale’s obtained total score was 38.17 ± 6.19 (Table 4). These results are interpreted as the items in the scale having high validity, distinguishing young adults in terms of methodological competencies, and being items intended to measure the same behavior [25].

**Table 4:** Item-total correlations of SRHSSS, t-test values for 27% lower–upper group difference

SRHSSS Items	Item-Total Correlations^1^	T test values (%27 lower–upper)^2^
1. SRH includes the social, physical, emotional and mental well-being of the individual and their partner	0,565	-3,944*
2. SRH is important for the general health, well-being and quality of life of individuals	0,596	-4,241*
3. As a young adult, I think it would be helpful for me to acquire basic information about SRH	0,604	-4,554*
4. As a young adult, I think it is important to get services from an official institution regarding my SRH problems	0,596	-5,031*
5. I think that the most accurate and reliable information on SRH can be given by the Ministry of Health and relevant official institutions	0,516	-4,554*
6. Every individual, male and female, young and old, should benefit from the consultancy services to be created or available in official institutions on SRH	0,566	-4,095*
7. Having information about SRH provides awareness about regular health checks	0,616	-5,031*
8. Having knowledge about SRH protects the person from risky situations such as random unsafe sexual intercourse	0,598	-4,390*
9. Unwanted and adolescent pregnancies can be prevented with SRH education	0,613	-4,554*
10. Having knowledge about SRH protects people from sexually transmitted diseases such as HIV and AIDS by providing awareness	0,623	-5,313*
11. Having knowledge about SRH enables to use effective and healthy prevention methods	0,598	-4,537*
12. I feel ready for information and discussion about SRH	0,683	-25,566*
13. Regardless of the gender of the health personnel, I feel comfortable talking about my SRH problems with the health personnel	0,757	-37,174*
14. I do not think that I would be ashamed if I received information about SRH from the SRH unit and/or health personnel in an official institution	0,768	-40,213*
15. I do not think that I will be blamed when I receive information about SRH from the SRH unit in an official institution	0,771	-40,967*
16. Regardless of their age, I feel comfortable talking about SRH issues with healthcare professionals	0,763	-36,401*
17. As a young adult, when I talk about SRH issues, I don't think the medical staff will be offended	0,760	-37,044*
18. Those who are faced with unwanted health problems by obtaining information on SRH informally are individuals who cannot receive age-appropriate education in the family and in formal education	0,461	-5,216*
19. In order to protect and maintain SRH, there is a need for adequate and accurate, age-appropriate education on SRH	0,501	-5,148*
20. I think that SRH education that can be planned by an official institution during the school years of young adulthood will prevent students from accessing unreliable sources and being harmed	0,519	-5,148*
21. With age-appropriate SRH education, individuals can make respectful and conscious choices by developing healthy behaviors	0,503	-5,289*
22. As a young adult, I think that a live support line for SRH counseling should be established by an official institution, rather than resorting to unreliable sources on the internet	0,419	-6,034*
23. I think that I can get adequate and reliable answers to my questions through the SRH consultancy support line or live interview to be provided on the internet by an official institution	0,410	-5,531*
**Total Score**	38.17 ± 6.19	

^1^*n* = 458, ^2^n_1_n_2_ = 124, *p < 0.001

## Dıscussıon

In this study, a valid and reliable measurement tool was developed to measure SRH service seeking, and the features of this tool were explained.

In the exploratory factor analysis conducted to examine the construct validity of SRHSSS, a four-factor structure was obtained that explained 89.45% of the total variance. The factor loadings of the scale items were found to vary between 0.78–0.97. Büyüköztürk states that a factor load value of 0.45 and above is a good criterion for selection, since the factor load values of the items are higher than 0.45, which means that these components collectively measure an idea, structure and factor. In addition, it is advised that there should be a minimum 0.10 difference between the highest load value of an item in the factors and the highest load value after this value.' According to the explanation in the form, it was possible to select items measuring the same construct in this study. The load difference between the factors of an item is higher than 0.10. This shows that this study achieved a good factorization. The investigation conducted to ascertain if the sample size was adequate for factor analysis revealed that the sample size was adequate because the KMO value was above 0.60. Büyüköztürk (2021) also stated that “If there is a cluster of items that are highly correlated with a factor, this finding means that those items together measure a concept-structure-factor. Regardless of sign, a load value of 0.66 and above is considered high; The range of loads between 0.30 and 0.59 is known as the medium magnitude range and is taken into account in subtracting the variable” (Table 1) [25].

In this study, the internal consistency coefficient for the whole of SRHSSS and its sub-dimensions was found to be 0.90 and above. According to Tavşancıl, one of the approaches used to determine the reliability or consistency of the measurements is the re-test method. The same measurement tool is applied to the same group after a certain period of time and the relationship between the measurements is found. This reliability coefficient is also called the continuity or stability coefficient and determines the extent to which the measurement tool measures the permanent characteristics of the individual.' In this study, the re-test method was used to evaluate the reliability of SRHSSS and at a high-level positive relationship was found (*r* = 0.713, *p* < 0.001). Tavşancıl stated, “To determine that a scale is invariant over time, the calculated correlation coefficient should be positive and high, and this limit is at least 0.70 for scales.” In this study, it can be said that SRHSSS has a high level of reliability and resolutely measures the service-seeking status of young adults [26].

For determining the total score discrimination of SRHSSS items, item-total correlations were investigated and 27% lower–upper group comparisons were made. According to Büyüköztürk, ‘Item-total correlation, which demonstrates how each item in a measuring instrument displays comparable behaviors, explains the link between the scores gained from the test items and the overall score of the test. The item-total correlation should be strong and positive in this situation. Items with a score of 0.30 or above are seen to have enough representation power of the scale when interpreting the item-total correlation.' In this study, it was found that the overall item-total correlations in the SRHSSS ranged between 0.41–0.77 and the 't' values were significant (*p* < 0.001). As a result, it is possible to say that the SRHSSS items illustrate similar behaviors, distinguish the participants in terms of SRH service-seeking situations, and thus have a high internal consistency [25].

In a study which is conducted with adolescents and young adults, a scale has been developed which has 23 questions and seven subscales. All subdimensions had good internal consistency reliability (Cronbach’s alpha scores > 0.7) and all items had rotated factor loadings > 0.5. Additionally, it is indicated that additional measures are necessary for this group because of their unique circumstances and life stage, which typically involve frequent changes in sexual partners and parental involvement in decision-making [3]. In another study by Cleland (2001), researchers designed a measurement tool to document knowledge, beliefs, behaviors, and outcomes and also to assess young people’s needs and problems in the field of SRH [16].

The implementation of SRHSSS in young adults or in different age groups will be able to measure the service-seeking of individuals in this regard, and accordingly, it will enable studies to facilitate access to health services. It is thought that SRHSSS has sufficient validity and reliability to be used in researches in the field of SRH.

### Limitations

The high factor loading related to subscale 1 (Individual Considerations in Service Seeking) has been determined as limitation in this study. In addition the lower values on test- retest for the sub-scales have been evaluated as limitation for this study.

## Conclusıons

Since SRH is an important issue for young adults, it is necessary to understand the thoughts, attitudes and perceptions of this age group on the subject. The SRHSSS is a scale with sufficient validity and reliability to identify and evaluate young adults' thoughts, attitudes, and perceived barriers to accessing services regarding SRH.

## Data Availability

The datasets used and/or analyzed during the current study are available from the corresponding author on reasonable request.

## Abbreviations

AIDS: Acquired Immunodeficiency Syndrome
HIV: Human Immunodeficiency Virus
KMO: Kaiser-Mayer-Olkin
SRH: Sexual and Reproductive Health
SRHSSS: Sexual and Reproductive Health Service Seeking Scale
STDs: Sexually Transmitted Diseases
WHO: World Health Organization
